# *Chlamydia trachomatis* Incidence and Re-Infection among Young Women – Behavioural and Microbiological Characteristics

**DOI:** 10.1371/journal.pone.0037778

**Published:** 2012-05-25

**Authors:** Jennifer Walker, Sepehr N. Tabrizi, Christopher K. Fairley, Marcus Y. Chen, Catriona S. Bradshaw, Jimmy Twin, Nicole Taylor, Basil Donovan, John M. Kaldor, Kathleen McNamee, Eve Urban, Sandra Walker, Marian Currie, Hudson Birden, Francis Bowden, Jane Gunn, Marie Pirotta, Lyle Gurrin, Veerakathy Harindra, Suzanne M. Garland, Jane S. Hocking

**Affiliations:** 1 CERSH Melbourne Medical School, University of Melbourne, Victoria, Australia; 2 Centre for Women's Health, Gender and Society, Melbourne School of Population Health, University of Melbourne, Victoria, Australia; 3 Molecular Microbiology Department, the Department of Microbiology and Infectious Diseases, the Royal Women's Hospital, Melbourne, Australia; 4 Melbourne Sexual Health Centre, Carlton Victoria, Australia; 5 Sexual Health Unit, School of Population Health, University of Melbourne, Victoria, Australia; 6 Department of Epidemiology and Preventive Medicine, Monash University, Melbourne, Victoria, Australia; 7 Kirby Institute, University of New South Wales, and Sydney Sexual Health Centre, Sydney Hospital, Sydney Australia; 8 Family Planning Victoria, Melbourne, Australia; 9 Monash Medical Centre, Department of Obstetrics and Gynaecology, Clayton, Victoria, Australia; 10 Australian National University, Canberra, Australian Capital Territory, Australia; 11 University Centre for Rural Health, and Sydney Institute for Emerging Infectious Diseases & Biosecurity, Sydney School of Public Health, The University of Sydney, Lismore, New South Wales, Australia; 12 Primary Care Research Unit, Department of General Practice, University of Melbourne, Victoria, Australia; 13 Centre for Molecular, Environmental, Genetic and Analytic Epidemiology, School of Population Health, University of Melbourne, Victoria, Australia; 14 St Mary's Hospital, Portsmouth, United Kingdom; 15 Department of Obstetrics and Gynaecology, University of Melbourne, Victoria, Australia; University of California Merced, United States of America

## Abstract

**Background:**

This study aimed to estimate rates of chlamydia incidence and re-infection and to investigate the dynamics of chlamydia organism load in prevalent, incident and re-infections among young Australian women.

**Methods:**

1,116 women aged 16 to 25 years were recruited from primary care clinics in Australia. Vaginal swabs were collected at 3 to 6 month intervals for chlamydia testing. Chlamydia organism load was measured by quantitative PCR.

**Results:**

There were 47 incident cases of chlamydia diagnosed and 1,056.34 person years of follow up with a rate of 4.4 per 100 person years (95% CI: 3.3, 5.9). Incident infection was associated with being aged 16 to 20 years [RR = 3.7 (95%CI: 1.9, 7.1)], being employed [RR = 2.4 (95%CI: 1.1, 4.9)] and having two or more new sex partners [RR = 5.5 (95%CI: 2.6, 11.7)]. Recent antibiotic use was associated with a reduced incidence [RR:0.1 (95%CI: 0.0, 0.5)]. There were 14 re-infections with a rate of 22.3 per 100 person years (95%CI: 13.2, 37.6). The median time to re-infection was 4.6 months. Organism load was higher for prevalent than incident infections (p<0.01) and for prevalent than re-infections (p<0.01).

**Conclusions:**

Chlamydia is common among young women and a high proportion of women are re-infected within a short period of time, highlighting the need for effective partner treatment and repeat testing. The difference in organism load between prevalent and incident infections suggests prevalent infection may be more important for ongoing transmission of chlamydia.

## Introduction

*Chlamydia trachomatis* (chlamydia) is the most common bacterial sexually transmitted infection (STI) worldwide. Both men and women can be infected with chlamydia causing urethritis and epididymitis in men and cervicitis in women [1], [2], [3]. If left untreated, chlamydia can lead to pelvic inflammatory disease (PID), tubal factor infertility and ectopic pregnancy in women [2], [4].

Chlamydia incidence estimates among women vary according to the population studied and range from between 4.9% per year in general practice populations in the UK [5] and 34 per 100 person-years in high-risk adolescents in the US [6]. Re-infection estimates also vary, but have been found to range between 4 to 51% per year [7], [8], [9], [10] in community-based studies and up to 84% in high-risk adolescents [6].

There are inconsistent findings about clinical and epidemiological associations with organism load. Some research reports associations between increased organism load and younger age [11], transmissibility and persistence of infection [12] and the risk of developing chronic sequelae [13], with more recent research finding no such associations [14], [15]. However, most studies have investigated organism load using a cross-sectional study design where it is not possible to determine when the infection was acquired [11], [13], [15], [16]. This highlights the need for longitudinal studies of chlamydial load in incident infection to further our understanding of whether organism load may be associated with chlamydia transmission.

We describe here the results of a cohort study of young women that aimed to estimate rates of chlamydia incidence and re-infection and to investigate the dynamics of chlamydia organism load in prevalent, incident and re-infection.

## Methods

### Recruitment

Women aged 16 to 25 years were recruited from general practice, family planning and sexual health clinics in Victoria, New South Wales and the Australian Capital Territory, three jurisdictions in South-Eastern Australia. The methods have been previously described [17]. In brief, written informed consent was obtained by all participants in the study. All eligible women were assessed for competency by research staff prior to being invited into the study. The research staff worked closely with the clinical staff to ensure only competent women were approached. All ethics committees approved the inclusion of participants over the age of 16 without parental or guardian consent. Women were recruited when they attended the clinic for any reason during the recruitment period and were eligible if they had ever had vaginal sex with a man, were not pregnant, could comprehend written English and were contactable by post during the 12-month period of the study. Women were tested at the time of recruitment (baseline) and at 6 and 12 months; women who tested positive at any stage were also re-tested three months after a positive test. All follow-up testing was conducted using self-collected vaginal swabs sent through the mail.

### Testing, organism load and serovar determination

#### Chlamydia testing

At the time of recruitment, women provided a first-pass urine specimen or self-collected vaginal swab which was tested by their clinician's pathology provider using nucleic acid amplification techniques (NAAT). They also provided an additional self collected flocked swab (Copan, Brescia, Italy) at baseline which was sent to the Molecular Microbiology Laboratory at the Department of Microbiology and Infectious Diseases, at the Royal Women's Hospital, Melbourne, Australia for quantification of chlamydial load and serovar determination should the woman be diagnosed with chlamydia at baseline. At each point in follow up, women were tested using a self-collected flocked swab that was sent through the Australian postal system to the laboratory at the Royal Women's Hospital. Chlamydia testing was performed using the Cobas TaqMan CT assay (Roche Applied Science) as per the manufacturer's instructions with 25 µl of extracted DNA using amplification and detection on the Cobas TaqMan 48 System (Roche Applied Science).

#### Quantification of organism load

Once detected, quantification of chlamydial load was determined by a quantitative PCR (qPCR) system targeting the omp1 gene using published methodology [18]. The chlamydia load in each sample was quantified by comparing the crossing-threshold of each sample to the crossing-threshold of a standard curve constructed by amplifying different known copy numbers of the omp1 gene. This method also determined whether any mixed infections were present, and identified the chlamydia serovar(s) of each infection through a series of qPCR assays using serovar-specific probes. Beta globin gene qPCR was used to assess sample adequacy as well as to measure sampling variability between participants and swabs by correlation with the number of eukaryotic cells collected. The quantity of chlamydia was divided by the number of eukaryotic cells and expressed as the number of organisms present per 100 eukaryotic cells [14].

#### Serovar detection

Confirmation of each chlamydia serovar, and detection of genotypic variants was determined by DNA sequencing across all four variable domains of the *omp1* gene that encodes for the antigenic major outer membrane protein as previously described [19].

### Management of participants

Women who tested positive at baseline were managed by their clinician. The research team provided free treatment of 1 gram stat of oral azithromycin. Clinicians were given an education pack with the latest treatment guidelines [20], partner notification support and a telephone number they could call to discuss clinical management of STIs with a sexual health physician [17]. The research team contacted each clinic after recruitment to confirm that the azithromycin was prescribed to women testing positive for chlamydia at baseline. If a participant tested positive during the course of the study, she was contacted by the research team who coordinated a telephone consultation with a sexual health physician. Azithromycin was sent to the participant free of charge along with partner notification letters and information sheets about chlamydia. When appropriate, treatment was provided to the woman's current sex partner. If the sexual health physician decided more clinical intervention was required, arrangements were made so that the woman had a face-to-face consultation with a clinician.

### Definitions

**Figure 1:**
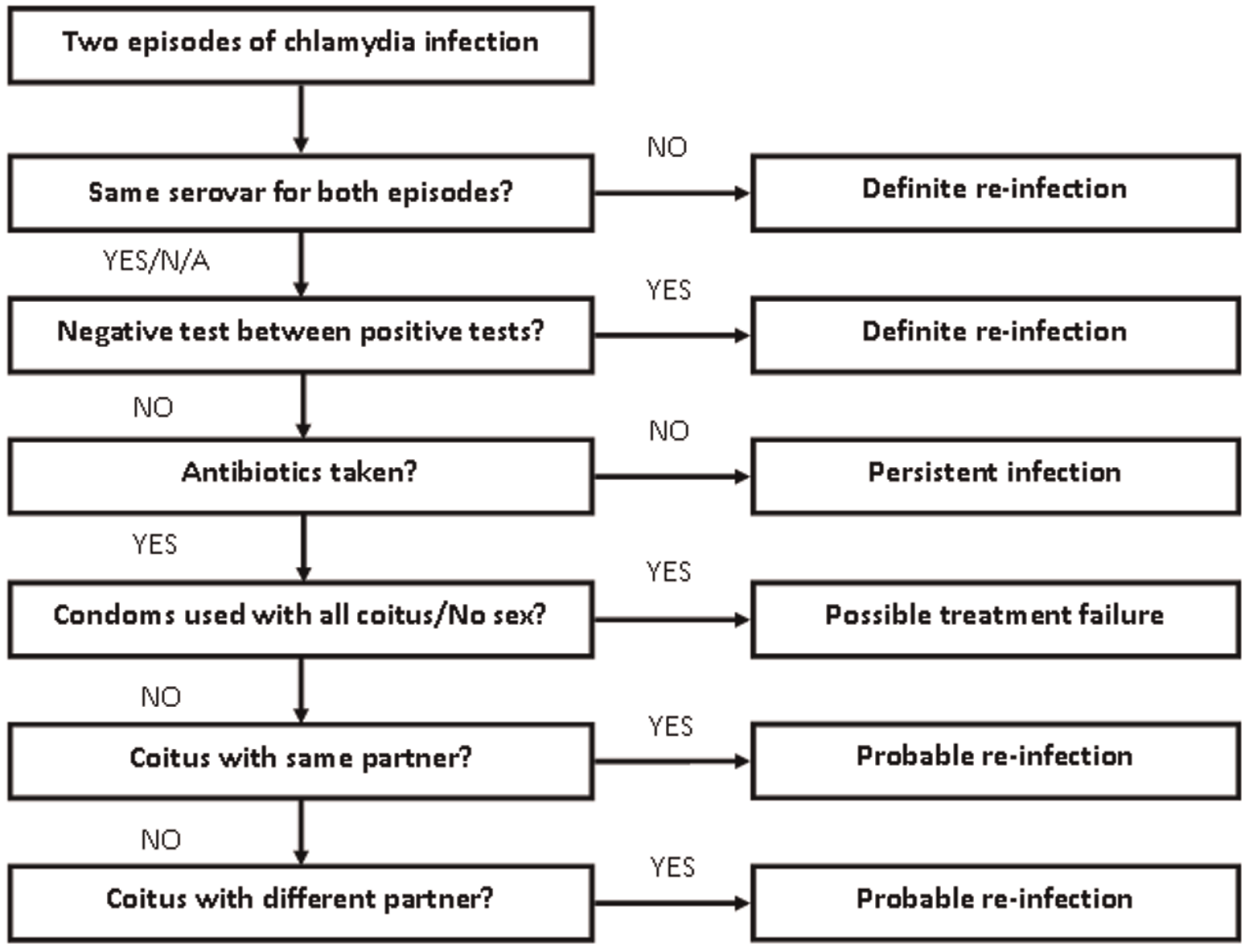
Algorithm to differentiate between chlamydia re-infection, treatment failure and persistent infection [adapted from Batteiger et al (2009)] [**6**]. N/A = Serovar result not available.

A *prevalent infection* was defined as a positive chlamydia result at the time of recruitment (baseline). An *incident infection* was defined as a positive chlamydia result diagnosed on a follow up test during the study period. Women were classified as having a *re-infection* if they tested positive for chlamydia again during follow up after a previous positive result earlier in the study, with or without a negative test in between positive test results. The subsequent positive results were defined as *re-infections*. Any re-infections diagnosed were also considered incident infections. To differentiate between *chlamydia re-infection, treatment failure* or *persistent infection*, we used a modified version of the chlamydia re-infection algorithm developed by Batteiger and colleagues (Figure 1) [6]. If a woman had two infections with different genotypes, then the second infection was considered a re-infection. If there had been a confirmed negative test between two positive results, the second infection was also considered a re-infection. If women had taken the correct antibiotics but had unprotected sex with her current partners or new partners, the second infection was also considered a re-infection. *Treatment failure* was defined as a positive chlamydia result following treatment with azithromycin if a woman reported she had either abstained from sex between the two tests or always used condoms with sex. An infection was defined as *persistent* if the woman had two consecutive positive chlamydia test results and was not treated following the first positive result.

### Data collection

At recruitment, women were asked to complete a self-administered paper-based questionnaire which collected demographic data. Paper-based questionnaires were also sent every three months and asked about sexual behaviour (number of sex partners, new sex partners and condom use), recent antibiotic and contraceptive use, and any pregnancies including termination and/or miscarriages. It also included questions about the presence of any recent genital symptoms, including abnormal vaginal discharge, abnormal vaginal odour, dysuria, abdominal pain and abnormal vaginal bleeding.

### Statistical methods

Calculations assuming a design effect of 2 indicated that a sample size of 1,000 would be sufficient to generate a 95% confidence interval incidence of 2.8 to 6.2 events per 100 person-years if the estimated rate is 4.5 events per 1000 person-years.

Chlamydia incidence and re-infection rates and 95% confidence intervals (95% CIs) based on the estimation of robust standard errors were calculated using Poisson regression. The rate of re-infection was calculated using only data from those who tested positive at least once during the study and had two or more tests. The Kaplan-Meier method was used to estimate the cumulative risk over time (equivalently the probability of remaining infection-free to any given time) of incident chlamydia infection or re-infection after initial infection. Follow-up was censored at 18 months after enrolment. The association between demographic, behavioural and clinical factors and the chlamydia incident or re-infection was investigated using a discrete-time version of the proportional-hazards regression model described by Carlin et al [21] from which rate ratios and robust standard errors were generated. For the purpose of the Kaplan Meier and proportional hazards regression modeling, re-infections were also considered as incident infections and included in the analysis of incident infections.

Factors associated with infection risk in a univariate analysis or that were clinically important in the view of the investigators were included in the multivariate model; if two factors were highly correlated, only one was included in the model.

The chlamydia organism load values were logarithm transformed for the purpose of analysis. Box plots were used to display the interquartile range, median value and overall range of organism load values. For the purpose of the box plots, data were stratified by infection type – prevalent, incident (where re-infections are excluded) and re-infection. T-tests or Mann-Whitney U-tests were used to compare organism load between groups where appropriate. Data were analysed using STATA version 11.1 [22].

Ethics approval to conduct this study was obtained from ten Human Research Ethics Committees throughout Australia.

## Results

### Sample characteristics

A total of 1116 women participated in the study with a recruitment response rate of 66%. The retention in the study over the 12 month period was 79% (877 women). There were 2,937 chlamydia tests conducted throughout the study. Overall, 738 women (66%) were recruited from general practice clinics and 378 (34%) were recruited from sexual health and family planning clinics. There were 55 cases of chlamydia diagnosed at the time of recruitment with a chlamydia prevalence of 4.9% (95% CI: 3.7%, 6.4%).

### Chlamydia incidence

**Figure 2:**
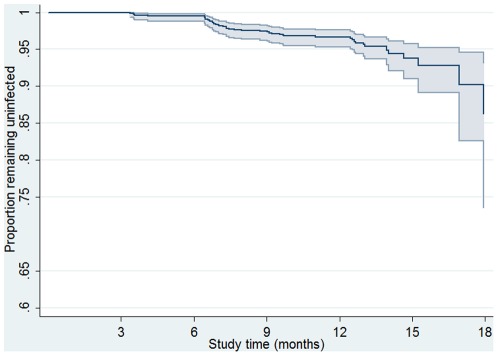
Kaplan Meier curve showing proportion remaining uninfected with incident chlamydia infection over time among a cohort of sexually active 16 to 25 year old women.

During the follow-up period, there were 47 cases classified as incident infections and 1056.34 person years of follow up with an incidence rate of 4.4 per 100 person-years (95% CI: 3.3, 5.9). Six women were diagnosed with two separate incident infections during the study period and 35 with a single incident infection. Chlamydia incidence was greatest 6 to 9 months after recruitment with 25 incident cases diagnosed during this time [rate = 9.2 per 100 person-years (95%CI: 6.2, 13.7)] (Figure 2). Chlamydia incidence was similar between women recruited from general practice clinics and those recruited from sexual health or family planning clinics [4.6 (95%CI: 3.3, 6.5) versus 4.1 (95%CI: 2.4, 7.0) per 100 person-years (p = 0.68)].

Univariate analysis found that being aged 16 to 20 years [RR = 3.5 (95%CI: 1.8, 6.8)], being less well educated [RR = 3.0 (95%CI: 1.4, 6.5)] and having two or more new sex partners [RR:4.8 (95%CI:2.4, 9.8)] were associated with incident chlamydia. Recent antibiotic use [RR:0.1 (95%CI: 0.0, 0.6)] was associated with reduced incidence. Multivariate analysis found that being aged 16 to 20 years [RR = 3.7 (95%CI: 1.9, 7.1)], being employed [RR = 2.4 (95%CI: 1.1, 4.9)] and having two or more new sex partners [RR = 5.5 (95%CI: 2.6, 11.7)] were associated with an increased incidence of chlamydia. Recent antibiotic use [RR:0.1 (95%CI: 0.0, 0.5)] was associated with a reduced incidence. Education level was highly correlated with age (p<0.01) and excluded from the model. Use of condoms, previously having chlamydia, and other demographic characteristics were not associated with incident infection (Table 1).

**Table 1:** Demographic and behavioural factors associated with chlamydia incident and re-infection among a cohort of sexually active 16 to 25 year old women.

	Incident infection*			Re-infection		
Variable	Incidence per 100 person years	Unadjusted rate ratio (95% CI^a^)	Adjusted rate ratio^b^ (95% CIa)	Re-infection rate per 100 person years	Unadjusted rate ratio (95% CI^a^)	Adjusted rate ratio^c^ (95% CIa)
**Age**						
16 to 20	7.7	3.5 (1.8, 6.8)	3.7 (1.9, 7.1)	31.3	3.0 (0.8, 10.5)	3.1 (0.9, 11.5)
21 to 25 (referent)	2.1	1.0	1.0	10.8	1.0	1.0
**Australian born**						
No (referent)	0.9	1.0		0.0	N/A	N/A
Yes	5.1	5.6 (0.8, 40.1)		23.6		
**Area of residence**						
Rural (referent)	4.4	1.0		15.2	1.0	1.0
Metropolitan	4.5	1.1 (0.6, 2.0)		30.2	2.0 (0.6, 6.9)	2.2 (0.6, 7.7)
**Education**						
Secondary school only	6.5	3.0 (1.4, 6.5)		23.9	1.2 (0.4, 3.7)	1.2 (0.4, 4.2)
Tertiary/further education (referent)	2.1	1.0		20.3	1.0	1.0
**Employment**						
Unemployed/Not working(referent)	3.3	1.0	1.0	12.7	1.0	1.0
Employed	5.3	1.6 (0.8, 3.3)	2.4 (1.1, 4.9)	29.0	2.3 (0.5, 10.8)	2.3 (0.5, 10.6)
**Clinic type**						
Sexual health/family planning clinic (referent)	4.1	1.0	1.0	28.1	1.0	N/A
General practice	4.6	1.1 (0.5, 2.3)	1.7(0.8, 3.8)	17.5	0.6 (0.2, 2.0)	
**Number of new partners since last follow up**						
0 (referent)	2.4	1.0	1.0	32.1	1.0	1.0
1	8.1	3.2 (1.6, 6.8)	3.1 (1.5, 6.7)	6.7	0.2 (0.1, 1.6)	0.2 (0.1, 1.7)
2+	14.3	4.8 (2.4, 9.8)	5.5 (2.6, 11.7)	14.8	0.5 (0.1, 1.9)	0.5 (0.1, 2.1)
**Recently had antibiotics**						
No (referent)	5.4	1.0	1.0	23.5	1.0	1.0
Yes	0,5	0.1 (0.0, 0.6)	0.1 (0.0, 0.5)	15.3	0.6 (0.1, 4.4)	0.7 (0.1, 4.4)
**Use of condoms**						
No (referent)	3.7	1.0		21.8	1.0	1.0
Yes	5.5	1.6 (0.9, 3.0)		24.2	1.1 (0.3, 3.7)	1.2 (0.3, 4.2)
**Previous positive ** ***C. trachomatis*** d						
No (referent)	4.4	1.0				
Yes	4.4	1.0 (0.3, 3.0)				

* **Analysis of incident infections includes re-infection.**

a **= confidence interval;**

b **= adjusted for age, employment, clinic type, number of new partners and recent antibiotic use;**

c **= adjusted for clinic type;**

d **= previous positive chlamydia test reported on questionnaire or diagnosed at time of recruitment into the study.**

### Re-infection

**Figure 3:**
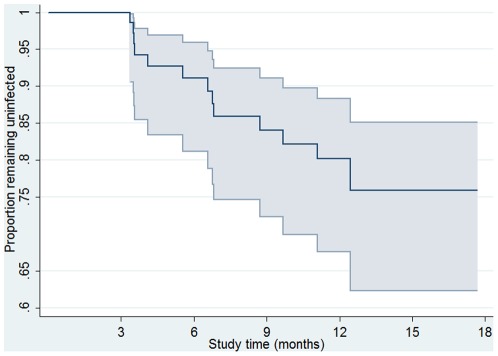
Kaplan Meier curve showing proportion remaining free from chlamydia re-infection over time among a cohort of sexually active 16 to 25 year old women.

There were 81 women who were at risk of re-infection during the study and contributed 62.7 years of follow-up. There were 14 re-infections of which seven were considered ‘definite’ re-infections in women who had had either a negative test in between two positive tests (four cases) or different serovars for each test (three cases). The other seven were ‘probable’ re-infections in women who had had unprotected sex in between positive results. Three women had two episodes of re-infection and eight had one episode of repeat infection. The overall re-infection rate was 22.3 (95%CI: 13.2, 37.6) per 100 person-years and the cumulative risk of re-infection during the study period was 20.3% (95%CI: 11.6, 31.7). The median time to re-infection was 4.6 months with 50% of re-infections acquired between 3.5 and 6.6 months after initial infection (Figure 3).

There were no associations found between re-infection and participant characteristics (Table 1).

### Persistent infections, treatment failure and ‘false positives’

One persistent infection was diagnosed in a woman who failed to take the prescribed antibiotics between two positive results of the same serovar. One possible case of treatment failure was identified in a woman who stated she had always had protective sex with condoms and her partner had been treated concurrently. One false positive was found in a participant who tested positive at follow-up despite taking antibiotics and having no risk of re-exposure. This woman was sent another swab that tested negative, and we concluded from her low risk of re-infection that her first test had been a false positive result. She was prescribed azithromycin as a precaution.

### Organism load by infection type

**Figure 4:**
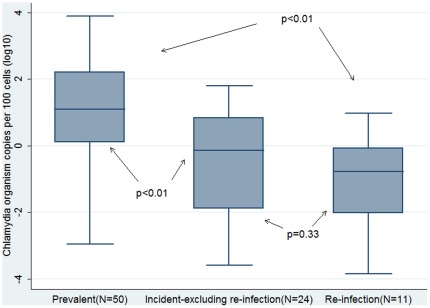
Comparison of chlamydia organism load for prevalent versus incident (excluding re-infection) and re-infection among a cohort of sexually active 16 to 25 year old women [shaded box = interquartile range; black line in box = median value; T bars = range of values].

Organism load was able to be measured for 50 (91%) prevalent infections, 24 (73%) incident (excluding re-infections) infections and 11 (79%) re-infections. Organism load was significantly higher for the 50 prevalent infections than for the 24 incident infections (excluding re-infections) (p<0.01) or the 11 re-infections (p<0.01). Organism load was not different between the 11 re-infections and the 24 incident infections (excluding re-infections) (p = 0.33) (Figure 4).

### Organism load for re-infections

For those with a re-infection, organism load was significantly lower for the first repeat infection compared with the initial infection (p<0.01), but there was no difference in load between the first and second repeat infections (p = 0.49) (Table 2).

**Table 2:** Organism load per 100 cells during episodes of chlamydia re-infection* among a cohort of sexually active 16 to 25 year old women.

Patient ID^a^	Initial infection	1^st^ re-infection	2^nd^ re- infection	Serovar
1	558.9	33.4		E, F
2	49886.9	48.9		E,E
3	43.7	17.7		E,E
4	2156.7	1.2		E,E
5	13110.5	<0.01	22.4	E,E,E
6	3207.5	0.1		K,E
7	153.9	6.2	6.6	K,E,E
8	3706.9	0.4		F,E
9	789.5	372.7		E,E

* **The time interval between the diagnoses was 3 to 6 months.**

a **= Excludes 2 people with missing organism load results.**

**Figure 5:**
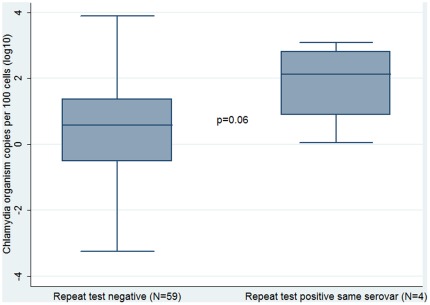
Comparison of chlamydia organism load at first diagnosis between women who had a negative repeat rest and women who had a positive repeat test of the same serovar among a cohort of sexually active 16 to 25 year old women [shaded box = interquartile range; black line in box = median value; T bars = range of values].

There were 63 women who tested positive during the study, had a follow up test and had organism load measured. This includes 37 women whose first positive result was detected at baseline and 26 women whose first positive result was detected on a follow up test. Among these 63 women, four (6%) were diagnosed with a repeat infection of the same serovar at their next test; the other 59 women tested negative (57) or tested positive with a different serovar (2).The organism load at the first test was higher for the four women who tested positive again with the same serovar than for those women who tested negative at their next test, although this was not a statistically significant difference (p = 0.06) (Figure 5).

Organism load was not associated with age (data not shown).

### Serovars

The chlamydia serovar was detected in the 39 of the 47 positive incident chlamydia samples; 60% were serovar E (n = 28), 11% were serovar F (n = 5), 6% were serovar D (n = 3), 4% were serovar G (n = 2) and 2% were serovar H (n = 1). The chlamydia serovars detected in the re-infection samples were primarily serovar E (n = 12, 86%), one was serovar D (7%) and one was serovar F (7%). Among prevalent infections, the majority were serovar E (Table 3). Overall, there were no associations observed between serovar and organism load or serovars and demographic characteristics.

**Table 3:** Chlamydia trachomatis serovars and genotypic variants detected in positive samples among a cohort of sexually active 16 to 25 year old women.

Chlamydia serovar	Genbank accession number	Prevalent infection N (%)	Incident infection (including re-infection) N (%)	Re-infection N (%)
D	HM230054	2 (3.6)	3 (6.4)	1 (7.1)
E	HM230055	27 (49.1)	28 (59.6)	12 (85.7)
E variant^a^	HM230056	1 (1.8)		
F	HM230057	12 (21.8)	5 (10.6)	1 (7.1)
G	HM230058	3 (5.5)	2 (4.3)	
G variant^b^	HM230059	2 (3.6)		
H	HM230060		1 (2.1)	
Ia	HM230061	1 (1.8)		
J	HM230062	1 (1.8)		
K	HM230063	3 (5.5)		
N/A^c^		3 (5.5)	8 (17.0)	
TOTAL		55	47	14

a **= E variant has 100% homology to Genbank sequence GU903922 (C. trachomatis strain 1969 from Australian male population);**

b **= G variant has 100% homology to Genbank sequence FJ261928 (G/IU-FW0267);**

c **= N/A: serovar unable to be determined.**

## Discussion

We present the incidence and re-infection rate data for a large cohort of young women and provide the first published quantitative data that investigates chlamydia organism load and its association with prevalent infections, incident infections and re-infections. We found very high re-infection rates of 22.3 per 100 person years and also found that organism load was higher in prevalent infections than in incident infections. These findings indicate prevalent infections maybe more important to ongoing transmission because of their higher organism load and chlamydia control strategies should include a focus on reducing both chlamydia prevalent infection and re-infection.

These data are the first chlamydia incidence and re-infection data for Australian women and show a chlamydia incidence of 4.4 per 100 person-years and a re-infection rate of 22.3 per 100 person-years which is comparable to other research findings in the UK [5]. We found that younger women (16 to 20 years old) and women who had had more sex partners during the study period were more likely to have an incident infection; this is also consistent with other studies [5], [23], [24]. Interestingly, we found that recent use of antibiotics was protective against an incident infection; this was also observed in a recently conducted survey of antenatal women in Australia [25] and raises the possibility that background antibiotic use in a population may have an impact on chlamydia transmission, particularly in Australia [26].

Our re-infection rate of 22.3 per 100 person-years is alarming, particularly considering the evidence suggesting that repeated infections markedly increase a woman's risk of PID and tubal damage [27], [28], [29], [30]. Australia is not alone with high re-infection rates, similarly high rates have been observed in the UK among women recruited from general practice and sexual health clinics [5] and in the USA among high risk adolescents [6]. We found that the median time until re-infection was 4.6 months. This provides further support for a test of re-infection at about 3 months which is currently recommended in Australia [20]. The high re-infection rates also highlight the importance of partner notification. Mathematical modelling of chlamydia transmission suggests that about 30% of current sex partners need to be treated in order to counterbalance the effect of re-infection [31]. Chlamydia control policy must include a focus on reducing re-infection through both increased re-testing at 3 months and improved partner notification. A recent systematic review found that mailed screening kits and reminder systems can be effective at increasing re-testing [32] both of which would be feasible in general practice with appropriate training and support.

The higher organism load in prevalent compared with incident infections including re-infections, is difficult to interpret because infections diagnosed at recruitment would have included both recently acquired and infections of over 12 months duration. However, it gives rise to a number of different hypotheses that may help explain this difference in organism load. Firstly, organism load may be lower with re-infections because past chlamydial infection confers some protective immunity and impacts on organism replication, reducing the organism load but not blocking chlamydial entry [14], [33]. This protective immunity hypothesis correlates well with the characteristics and mechanisms of partial protection defined in mouse and guinea pig models of genital infection [33]. However, proof of this hypothesis would require further longitudinal studies that include collection of comprehensive behavioural data to capture the duration of infection at recruitment, baseline and subsequent sampling of immunological markers together with serial sampling for incident infection and determination of organism load [34]. A second hypothesis is that organism load increases over time following infection. However, this hypothesis seems unlikely because chlamydia infection will naturally clear over time without treatment [35]. Further, mouse models have shown that chlamydial shedding decreases over time following infection [36], [37]. A third hypothesis is that lower organism load infections naturally clear more quickly than higher load infections and on this basis, prevalent infections represent a biased sample of higher organism load infections. This hypothesis is supported by mathematical models of chlamydia transmission.[38] Regardless of the mechanisms to explain the difference we observed in organism load, it is probable that infections of shorter infection duration with quantitatively less organism shedding are associated with decreased risk of transmission which suggests prevalent infections maybe more important to ongoing infection transmission. This highlights the importance of increasing screening coverage among the target population in chlamydia control programs. One other study has suggested that organism load decreases with subsequent infections, but this was based on only four participants [14], with our results providing further evidence for this finding.

Also, we found that women with higher organism loads were more likely to have a repeat infection of the same serovar at next test than women who did not have a repeat infection at their next test. While this was not a statistically significant result and based on only four cases, a p value of 0.06 does provide some weak evidence to warrant further discussion. It has been shown in trachoma treatment studies that azithromycin failure is more likely at higher organism loads and this raises the question of whether some repeat infections in our study could represent treatment failure rather than re-infection. Using our re-infection algorithm, we did identify one potential case of treatment failure (2%) which is lower than the 8% reported in similar studies. However, given the time interval between tests in our study (three to six months on average) it is likely that it was challenging for women to reliably report sexual exposure between tests reducing our ability to reliably differentiate between re-infection and treatment failure. In light of the increasing concern about the possibility of azithromycin treatment failure [6], [39], [40], [41], [42], further studies with larger sample sizes and more frequent sampling are necessary to explore this.

There were some limitations to our results. Firstly, our algorithm and classification of re-infection is partially dependent on self-reported sexual behaviour which can be influenced by social desirability bias. Increasingly specific molecular analysis such as the use of multi locus sequence typing (MLST) should help reduce this bias, although it will not eliminate it [43]. Secondly, the precision of our estimates of incident and re-infection rates and cumulative risk reflect the availability of event data, which was constrained by the frequency and timing of our specimen collection. Our analysis of re-infection and organism load was also limited by sample size. Thirdly, there are potential limitations with the organism load analysis as a result of not being able to analyse organism load on all the swabs. A greater proportion of incident infections had missing organism load data than prevalent infections. This is likely to be because of the reduced sensitivity of qPCR when the organism load is low [18]. However, there were no differences in participant characteristics including age or number of partners between those who had measurable organism load and those who did not. Variation in organism load may occur daily as the infection progresses or resolves and may also be affected by the state of the menstrual cycle [44]. However, it would not be possible to control for the menstrual cycle due to hormonal fluctuations that vary from one woman to another even at the same stage of the cycle, which is difficult to measure precisely [14]. To adjust for individual sampling variability, organism load was quantified relative to the number of eukaryotic cells per sample.

A recruitment response rate of 66% is very high for this type of study exploring socially sensitive material [45]. However, as we have described elsewhere, women who participated in this study were more likely to be Australian born, more well educated and reported a greater number of sex partners than the background Australian population of the same age [17], [46]. As a result, our study may not be representative of all Australian 16 to 25 year old women. Nevertheless, our retention rate of 79% over the duration of the study with negligible loss to follow up bias is a significant strength of our study, adding weight to our findings [17]. Further, the serovars detected in the cohort were consistent with previously published Australian and international data suggesting our results were broadly representative of the Australian population.

This is the first published cohort study of chlamydia incidence and re-infection among young women that has investigated organism load by infection type. We have shown that chlamydia is a common infection in young Australian women and considering the high proportion of women who are re-infected within a short period of time, innovative and effective strategies are needed to encourage increased re-testing following a positive diagnosis and improved partner notification. We also found that organism load in incident infection is less than prevalent infections suggesting that prevalent infection might be more important in the transmission of chlamydia. Further longitudinal studies that use serial collection of behavioral and biological data are necessary to investigate protective immunity and the responsible mechanisms.
